# Supplementary material for: Proof of concept for multiple nerve transfers to a single target muscle
Source: eLife. 2021 Oct 1;10:e71312. doi: 10.7554/eLife.71312 (PMC8530510; doi:10.7554/eLife.71312)
Supplement: Source code 1. — The respective headings for the code sections are highlighted using asterisks. [file elife-71312-supp1.zip › Syntax code for analysis.docx]

**SPSS 25 Syntax used for Muscle mass analysis:**

***create diagnostic scatterplots for assumptions***

GGRAPH

/GRAPHDATASET NAME="graphdataset" VARIABLES=control treated Treatment MISSING=LISTWISE

REPORTMISSING=NO

/GRAPHSPEC SOURCE=INLINE

/FITLINE TOTAL=NO SUBGROUP=NO.

BEGIN GPL

SOURCE: s=userSource(id("graphdataset"))

DATA: control=col(source(s), name("control"))

DATA: treated=col(source(s), name("treated"))

DATA: Treatment=col(source(s), name("Treatment"), unit.category())

GUIDE: axis(dim(1), label("control"))

GUIDE: axis(dim(2), label("treated"))

GUIDE: legend(aesthetic(aesthetic.color.exterior), label("Treatment"))

GUIDE: text.title(label("Grouped Scatter of treated by control by Treatment"))

SCALE: linear(dim(1), min(100), max(450))

SCALE: linear(dim(2), min(100), max(450))

SCALE: cat(aesthetic(aesthetic.color.exterior), include("1", "2"))

ELEMENT: point(position(control*treated), color.exterior(Treatment))

END GPL.

***Homogenity of regression slopes***

UNIANOVA treated BY Treatment WITH control

/METHOD=SSTYPE(3)

/INTERCEPT=INCLUDE

/CRITERIA=ALPHA(0.05)

/DESIGN=Treatment control Treatment*control.

***Univariate ANCOVA***

UNIANOVA treated BY Treatment WITH control

/METHOD=SSTYPE(3)

/INTERCEPT=INCLUDE

/SAVE=RESID ZRESID

/EMMEANS=TABLES(Treatment) WITH(control=MEAN) COMPARE ADJ(BONFERRONI)

/PRINT ETASQ DESCRIPTIVE HOMOGENEITY

/CRITERIA=ALPHA(.05)

/DESIGN=control Treatment.

***Testing for Normality***

EXAMINE VARIABLES=ZRE_1 BY Treatment

/PLOT BOXPLOT NPPLOT

/COMPARE GROUPS

/STATISTICS DESCRIPTIVES

/CINTERVAL 95

/MISSING LISTWISE

/NOTOTAL.

***Testing for homoscedasticity **

GGRAPH

/GRAPHDATASET NAME="graphdataset" VARIABLES=PRE_1 ZRE_1 Treatment MISSING=LISTWISE

REPORTMISSING=NO

/GRAPHSPEC SOURCE=INLINE

/FITLINE TOTAL=NO.

BEGIN GPL

SOURCE: s=userSource(id("graphdataset"))

DATA: PRE_1=col(source(s), name("PRE_1"))

DATA: ZRE_1=col(source(s), name("ZRE_1"))

DATA: Treatment=col(source(s), name("Treatment"), unit.category())

GUIDE: axis(dim(1), label("Predicted Value for treated"))

GUIDE: axis(dim(2), label("Standardized Residual for treated"))

GUIDE: axis(dim(3), label("Treatment"), opposite())

GUIDE: text.title(label("Simple Scatter of Standardized Residual for treated by Predicted ",

"Value for treated by Treatment"))

SCALE: cat(dim(3), include("1", "2"))

ELEMENT: point(position(PRE_1*ZRE_1*Treatment))

END GPL.

**SPSS 25 Syntax used for retrograde labeling analysis:**

*Nonparametric Tests: Independent Samples.

NPTESTS

/INDEPENDENT TEST (labeled_neurons) GROUP (Treatment)

/MISSING SCOPE=ANALYSIS USERMISSING=EXCLUDE

/CRITERIA ALPHA=0.05 CILEVEL=95.

***medians***

MEANS TABLES=labeled_neurons BY Treatment

/CELLS=COUNT MEDIAN.

***mean ranks***

NPAR TESTS

/K-W=labeled_neurons BY Treatment(0 2)

/MISSING ANALYSIS.

***boxplot***

EXAMINE VARIABLES=labeled_neurons BY Treatment

/PLOT BOXPLOT NPPLOT

/COMPARE GROUPS

/STATISTICS NONE

/CINTERVAL 95

/MISSING LISTWISE

/NOTOTAL.
