# Supplementary material for: Proof of concept for multiple nerve transfers to a single target muscle
Source: eLife. 2021 Oct 1;10:e71312. doi: 10.7554/eLife.71312 (PMC8530510; doi:10.7554/eLife.71312)
Supplement: Transparent reporting form [file elife-71312-transrepform1.docx]

***eLife’s* transparent reporting form**

We encourage authors to provide detailed information *within their submission* to facilitate the interpretation and replication of experiments. Authors can upload supporting documentation to indicate the use of appropriate reporting guidelines for health-related research (see [EQUATOR Network](http://www.equator-network.org/%20)), life science research (see the [BioSharing Information Resource](https://biosharing.org/)), or the [ARRIVE guidelines](http://www.plosbiology.org/article/info:doi/10.1371/journal.pbio.1000412) for reporting work involving animal research. Where applicable, authors should refer to any relevant reporting standards documents in this form.

If you have any questions, please consult our Journal Policies and/or contact us: [editorial@elifesciences.org](mailto:editorial@elifesciences.org).

**Sample-size estimation**

- You should state whether an appropriate sample size was computed when the study was being designed
- You should state the statistical method of sample size computation and any required assumptions
- If no explicit power analysis was used, you should describe how you decided what sample (replicate) size (number) to use

Please outline where this information can be found within the submission (e.g., sections or figure legends), or explain why this information doesn’t apply to your submission:

In the original design it was planned that three groups (single nerve transfer, double nerve transfer and triple nerve transfer) are compared with each other. The sample size of 15 per group was chosen to detect pairwise differences of 1.34 standard deviations in the primary endpoint with a power of 90% at a two-sided significance level of 5% by the Tukey-HSD-post-hoc procedure. For technical reasons the triple nerve transfer was not feasible, and we only performed the study for two groups, which increases the power because we could apply a simple two-groups t-test and no longer needed to adjust for multiple comparison. In fact, for the planned effect of 1.34 standard deviations we obtain a power of 94%. On the other hand, we were asked by the editor to include the potential effect of the control side in our analysis and therefore performed an ANCOVA, which will not particularly change our estimated power. We did not perform a two-way ANOVA and testing for the interaction effect, because this would not have considered the paired observations we have for each animal between the treatment and the control side. Please refer to section 2.1.

**Replicates**

- You should report how often each experiment was performed
- You should include a definition of biological versus technical replication
- The data obtained should be provided and sufficient information should be provided to indicate the number of independent biological and/or technical replicates
- If you encountered any outliers, you should describe how these were handled
- Criteria for exclusion/inclusion of data should be clearly stated
- High-throughput sequence data should be uploaded before submission, with a private link for reviewers provided (these are available from both GEO and ArrayExpress)

Please outline where this information can be found within the submission (e.g., sections or figure legends), or explain why this information doesn’t apply to your submission:

The total number of experiments is reported in 2.1. Experimental design and the exact number of examinations is depicted in the relevant methods paragraphs.

**Statistical reporting**

- Statistical analysis methods should be described and justified
- Raw data should be presented in figures whenever informative to do so (typically when N per group is less than 10)
- For each experiment, you should identify the statistical tests used, exact values of N, definitions of center, methods of multiple test correction, and dispersion and precision measures (e.g., mean, median, SD, SEM, confidence intervals; and, for the major substantive results, a measure of effect size (e.g., Pearson's r, Cohen's d)
- Report exact p-values wherever possible alongside the summary statistics and 95% confidence intervals. These should be reported for all key questions and not only when the p-value is less than 0.05.

Please outline where this information can be found within the submission (e.g., sections or figure legends), or explain why this information doesn’t apply to your submission:

Statistical analysis methods and tests are reported under 2.6 Statistical analysis. Exact p-values and effect sizes are reported in the description of figures 4 and 5, under 3.4.1 Comparison of reinnervated muscle mass and 3.4.2 Comparison of reinnervated and control muscle mass.

(For large datasets, or papers with a very large number of statistical tests, you may upload a single table file with tests, Ns, etc., with reference to sections in the manuscript.)

**Group allocation**

- Indicate how samples were allocated into experimental groups (in the case of clinical studies, please specify allocation to treatment method); if randomization was used, please also state if restricted randomization was applied
- Indicate if masking was used during group allocation, data collection and/or data analysis

Please outline where this information can be found within the submission (e.g., sections or figure legends), or explain why this information doesn’t apply to your submission:

Allocation of subjects is reported under 2.1. Experimental design while blinded data analysis is reported under 2.3. Behavioral evaluation and 2.4. Retrograde labeling.

**Additional data files (“source data”)**

- We encourage you to upload relevant additional data files, such as numerical data that are represented as a graph in a figure, or as a summary table
- Where provided, these should be in the most useful format, and they can be uploaded as “Source data” files linked to a main figure or table
- Include model definition files including the full list of parameters used
- Include code used for data analysis (e.g., R, MatLab)
- Avoid stating that data files are “available upon request”

Please indicate the figures or tables for which source data files have been provided:

Source data files for figures 4C and 5 including the corresponding SPSS Syntax used for analysis have been uploaded.
